# P04.40. Lifestyle therapy use in pediatric cancer survivors

**DOI:** 10.1186/1472-6882-12-S1-P310

**Published:** 2012-06-12

**Authors:** J Karlik, Y Bao, B Cheng, S Lees, D Ndao, E Ladas, K Kelly

**Affiliations:** 1 Columbia University Medical Center, Dept of Pediatric Oncology, New York City, USA; 2 Columbia University Medical Center, New York City, USA

## Purpose

Pediatric cancer survivors often experience late effects secondary to cancer therapy, but their choices for symptom management is largely unknown. Many of these late effects can be treated by lifestyle changes such as diet, exercise, and conventional supplements. Although the use of lifestyle therapies appears common in children and adolescents, including pediatric cancer survivors, the reasons for use and perceptions of efficacy have not been investigated.

## Methods

We report the results of a cross sectional survey investigating the prevalence of lifestyle therapy use, types and reasons for lifestyle therapy use, and determinants of lifestyle therapy use among survivors of childhood cancer.

## Results

One hundred fifty-five (95%) patients approached in person and 45 (34%) patients approached by mail consented to participate in the study. Twenty-four participants participated in an original survey and were longitudinally followed with a repeat survey approximately 10 years later. Average age of participants was 14.5 ± 6.2 years. The average time from completion of cancer treatment to survey administration was 4.5 ± 4.0 years. One hundred thirty-four (68%) made lifestyle modifications overall; 46% took multivitamin or conventional supplements, 45% used dietary changes, and 21% used exercise. Reasons for use included general health (87%) and specific symptoms/side effects (13%). Of specific reasons, general health and healing (31%) and fitness/weight control( 25%) were common. Sixty-one percent of participants thought their lifestyle change was very effective and 25% thought it was somewhat effective. Forty-three percent obtained lifestyle information from a family member/friend and 29% obtained information from their physician. Eighty-five percent of survivors disclosed the use of lifestyle modifications to their physician.

## Conclusion

Many pediatric cancer survivors use lifestyle therapies and believe that these changes are effective. Given the widespread use of lifestyle therapies in pediatric cancer survivors, research is needed on the efficacy of lifestyle modifications in treating symptoms/side effects and improving general health of pediatric cancer survivors.

